# 

**Published:** 1902-05-15

**Authors:** 


### ANNOUNCEMENTS.

### AMERICAN DENTAL SOCIETY OF EUROPE.

The next meeting of this society will be held in Stockholm, August 12th to 15th, inclusive. A cordial invitation is extended to the profession to meet with us.

This date will enable those attending the National, at Niagara, July 28th to 31st, to be present, by sailing via Hamburg after that meeting.

Owing to the heavy booking of steamer berths, it would be well for intending voyagers to secure their return passages in advance. The best way to reach Stockholm is via Hamburg. (1.) Travel tickets only for the journey. Hamburg, Kiel, Corsor, Copenhagen, Malmo, Stockholm and return to Hamburg by the same route, $32.58 per adult, first-class; $25.56 per adult, second-class. (2.) Travel tickets only for the route, Hamburg, Lubeck and steamer direct to Stockholm, returning to Hamburg by the same route, $20.46 per adult, first-class; $15.72 per adult, second-class.

In the case of route No. 1, the validity is forty-five days, and for route No. 2, the season. As much notice as possible should be given to secure accommodation. The times between Hamburg and Stockholm are as follows: Route No. 1, depart Hamburg 8:53 A. M. or 11:07 P.M.; arrive Copenhagen 6:54 P.M. or 10:05 A.M.; depart Copenhagen 7:45 P. M. or 11:15 A. M.; arrive Stockholm 11:25 A. M. or 6:45 A. M. Route No. 2, depart Hamburg 12:00 NOON, 2:00 P. M. or 3:40 P. M.; arrive Lubeck 1:21 P. M., 3:32 P. M. or 4:53 P. M.; depart Lubeck about 6:15 P. M. Wednesdays and Saturdays, occupying about forty-two hours, but times for the coming season, not yet fixed.

Any further information that may be desired can be obtained from Messrs. Cook & Sons, 261 and 262 Broadway, New York.

L. J. MITCHELL, *Hon. Sec.*,

39 Upper Brook St., London W.

### NATIONAL DENTAL ASSOCIATION.

As a result of the recent postal card vote, the date of the coming meeting of the National Dental Association has been changed to Monday, July 28th and to continue for four days thereafter.

A. H. PECK, *Recording Sec'y.*

### NEW JERSEY STATE DENTAL SOCIETY.

The Committee on Exhibits desires to announce that at the thirty-second annual meeting of the New Jersey State Dental Society, to be held as usual, in the "Auditorium," Asbury Park, July 16th, 17th and 18th, the large room which is especially adapted for exhibition purposes, will be devoted exclusively to the exhibits. Every advantage is here offered for a great display, with all the conveniences necessary for such an exhibition.

It is earnestly requested that those desiring space communicate with the Chairman at an early date.

FRANK L. HINDLE, New Brunswick, N. J.
*Chairman of the Committee on Exhibits.*

### DENTAL EXAMINATIONS.

The Board of Dental Examiners of Pennsylvania will conduct examinations in Philadelphia, June 24–27, 1902. For papers and further information address Hon. James W. Latta, Secretary Dental Council, Harrisburg, Pa.

G. W. KLUMP, *Secretary*,
Williamsport, Pa.

### THE MICHIGAN STATE DENTAL ASSOCIATION.

The Michigan State Dental Association will convene at Grand Rapids, June 9th, 10th and 11th. A good literary program is in preparation and an extension clinic is promised. It is expected that this location will attract a large number of dentists. The time for holding this meeting is favorable, no more accessible city could be selected, and there ought to be a large attendance.

### THE SOUTHWESTERN MICHIGAN DENTAL ASSOCIATION.

The Southwestern Michigan Dental Association held its spring semi-annual meeting at Buchanan, April 8th and 9th. There was an unusually large attendance, and there was more hard work and fun mixed together in two days than we are accustomed to witness in dental society meetings. This society has a large membership of boys both old and young and they seem to be able to get the most out of life that is possible. We shall publish the papers in the June issue. The fall meeting will be held at Three Rivers.

### THE CENTRAL MICHIGAN DENTAL ASSOCIATION.

The Central Michigan Dental Association was organized at Ionia, April 3rd. The society meets a long felt want, and starts out auspiciously with Dr. John J. Green, as President, and P. L. Campbell, of Ionia, as Secretary. If it lives to its promise we shall expect some good work from it.

### THE CHICAGO DENTAL SOCIETY.

The Chicago Dental Society, at its annual meeting April 1st, elected the following officers: President, Elgin MaWhinney; First Vice-President, H. J. Goslee; Second Vice-President, F. B. Noyes; Secretary, Winthrop Girling; Corresponding Secretary, C. S. Bigelow; Treasurer, E. R. Carpenter; Librarian, H. W. Sale; Member Board of Directors, Edmund Noyes; Board of Censors, W. V.-B. Ames, Chairman, C. N. Johnson, A. W. Harlan.

### WEST VIRGINIA BOARD OF DENTAL EXAMINERS.

The West Virginia Board of Dental Examiners will meet at Martinsburg May 21, 22 and 23, 1902, for the examination of candidates. The examination will be in writing, and will include anatomy, physiology, chemistry, histology, bacteriology, pathology, metallurgy, dental medicine, surgery, and operative and prosthetic dentistry, together with operations in the mouth.

All applications, together with the fee (ten dollars), should be sent in ten days before the examination. All applicants are expected to furnish their own instruments and material.

J. F. BUTTS, *Sec'y*,
Charleston, W. Va.

